# A Case Report of Fenestration Deformity of Extracranial Vertebral Artery and Literature Review

**DOI:** 10.1055/a-2572-7093

**Published:** 2025-05-10

**Authors:** Mingjuan Cao, Qun Qiang, Jia Liu, Miaomiao Zhang

**Affiliations:** 1 Department of Ultrasound Medical Imaging, Gansu Provincial Hospital of Traditional Chinese Medicine, Gansu, China

**Keywords:** artery/arteries, ultrasound, carotid arteries

## Abstract

**Background:**

Vertebral artery fenestration (VAF) is a rare anatomical variation of the vertebral artery that may affect the safety of clinical surgeries and interventional treatments. Although ultrasound is widely used to assess neck vessels, its application in diagnosing VAF has not been extensively investigated.

**Case Description:**

This article reports a case of an 82-year-old male patient who was diagnosed with VAF following a neck vessel ultrasound examination prompted by poor blood sugar control. The ultrasound revealed an abnormal course of the left vertebral artery, leading to the diagnosis of VAF.

**Conclusion:**

Ultrasound, with its noninvasive nature and real-time imaging capabilities, is a valuable initial screening tool for diagnosing VAF. Despite its limitations, its ability to identify vascular anomalies such as VAF highlights its critical role in early detection, potentially improving patient outcomes and guiding clinical decision-making.

## Introduction

Vertebral artery fenestration (VAF) is a rare anatomical variation of the vertebral artery. Studies based on autopsies and angiographic analysis indicate that this condition occurs in approximately 0.2 to 2% of individuals. 1 2 3 The characteristic of VAF is that the vertebral artery is segmentally divided into two parallel channels. These channels run along the course for a while and then merge into one channel again. 4 This feature is highly similar to the imaging features of vertebral artery dissection (VAD), thereby increasing the risk of misdiagnosis for VAF. 5 Ultrasound examination, due to its ability to directly visualize blood flow, has become the most routine and preferred method for examining cervical blood vessels. 6 However, there is a lack of research on the use of ultrasound in the diagnosis of VAF in existing domestic and international literature. Therefore, this study focused on a VAF case to explore the practical application value of ultrasound in the diagnosis of this disease.

## Clinical Data

**Fig. 1:**
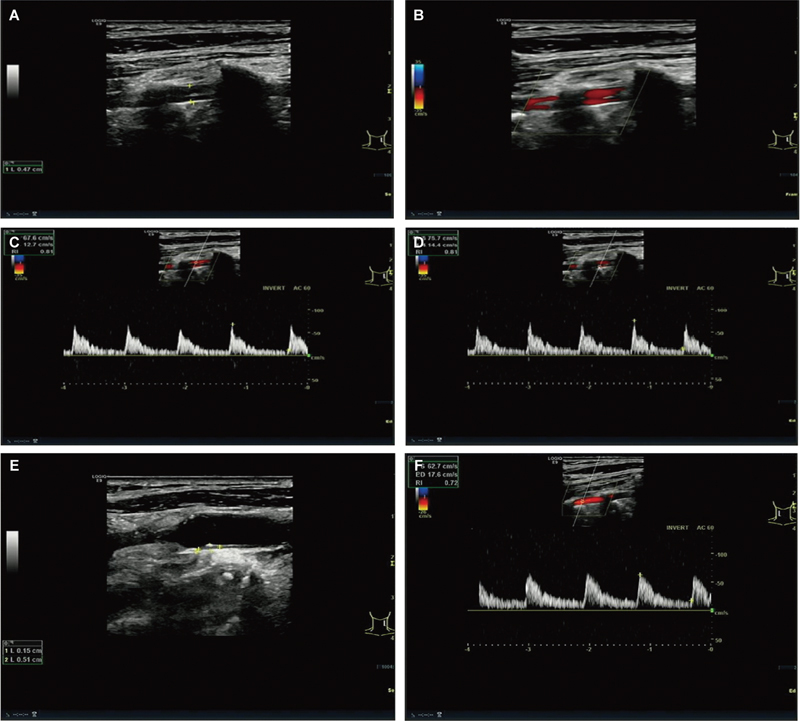
( **A** ) Two-dimensional image showing the vertebral artery fenestration. ( **B** ) Color Doppler shows that the blood flow direction and brightness of the two vertebral arteries are consistent. ( **C** ) Superficial quadratic artery spectrum. ( **D** ) Deep quadratic artery spectrum. ( **E** ) Color Doppler shows plaques on the posterior wall of the left common carotid artery bifurcation. ( **F** ) Blood flow spectrum of the right vertebral artery.

**Fig. 2:**
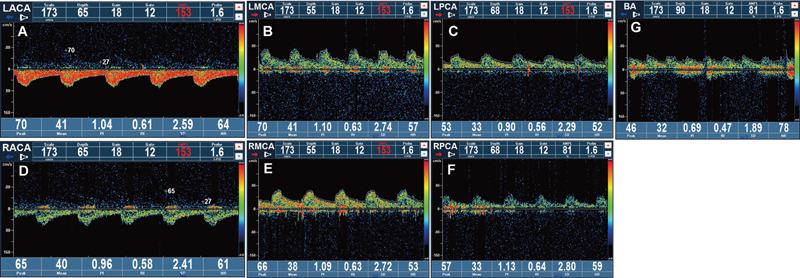
( **A–C** ) The ultrasonic Doppler spectrum of the left anterior cerebral artery, middle cerebral artery, and posterior cerebral artery. ( **D–F** ) The ultrasonic Doppler spectrum of the right anterior cerebral artery, middle cerebral artery, and posterior cerebral artery. ( **G** ) The ultrasound Doppler spectrum of the basilar artery. Among them, the color bar represents the blood flow velocity.

The patient, an 82-year-old male with no history of neck trauma, elevated blood sugar for more than 20 years, and poor blood sugar control for 1 week, was admitted to the hospital with “type 2 diabetic peripheral neuropathy and upper respiratory tract infection.” The patient's blood pressure was measured at 152/74 mmHg and fasting blood glucose concentration was >8.0 mmol/L. The results of routine color Doppler ultrasound examination of the neck vessels showed that the diameter of the left vertebral artery in the intervertebral space segment was 4.7 mm, and it was split into two unidirectional blood flows at the C3, C4, C5, and C6 intervertebral spaces. Both blood flows were well-filled, and their blood flow spectrum showed continuous forward blood flow throughout the cardiac cycle. The ultrasonic Doppler spectrum revealed a superficial blood vessel velocity of 67/12 cm/s and a deep blood flow velocity of 75/14 cm/s ( Fig. 1A–D ). The split blood vessels converged into a single blood flow in the V1 segment. In addition, plaque formation was visible on the posterior wall of the patient's left common carotid artery bifurcation ( Fig. 1E ). No obvious abnormalities were found in the right vertebral artery ( Fig. 1F ) and intracranial blood vessels ( Fig. 2 ). In summary, the ultrasound diagnosis was left VAF malformation.

## Discussion

VAF is a developmental abnormality during the embryonic period that begins around the 32nd day and develops by the fusion of the longitudinal neural artery with the anterior six pairs of intersegmental arteries in the neck until the 40th day. 7 If the primitive dorsal aortic arch does not completely regress and accompanies the intersegmental arteries, a VAF will form. According to the location of the deformity, VAF can be divided into intracranial, extracranial, and intracranial–extracranial types. According to the range of the deformity and imaging manifestations, fenestration can be divided into slit type (the range of the fenestration vessel: 2–3 mm), convex lens type (the range of the fenestration vessel: 5–10 mm), and duplication type (the range of the fenestration vessel: > 20 mm). This case belongs to the extracranial duplication type.

During an ultrasound examination, VAF should be distinguished from VAD. The imaging manifestations of VAD are mainly the presence of a torn intimal flap and a double lumen. This double lumen is that the same blood vessel is divided into a true lumen and a false lumen, and manifested as vascular stenosis, occlusion, and pseudoaneurysm. 8 The two parallel channels of VAF are both blood vessels with intact vascular walls, including the endothelium, muscularis, and adventitia. Its imaging manifestation is mainly the presence of segmental double lumens, which are two independent blood vessels. According to the patient's ultrasound imaging information, there are two blood vessels in the course of the left vertebral artery ( Fig. 1A ). Color Doppler measurement of the blood flow direction and brightness of these two blood vessels are consistent ( Fig. 1B ). Spectral Doppler showed that the superficial blood vessel velocities of the two vessels were similar to those of the deep blood velocity, which were 67/12 and 75/14 cm/s, respectively ( Fig. 1C, D ). Based on this judgment, we diagnosed the patient with VAF. The reasons are as follows: the true lumen and false lumen of VAD are generally one narrow and one wide, and their blood flow velocities are one fast and one slow. The ultrasound examination often shows that the true lumen blood flow is bright and the false lumen blood flow is dim. Second, during the dynamic tracking scan, VAD can display the site and range of the intimal tear, while VAF can display the vascular bifurcation and confluence.

There are some additional factors to consider in the diagnosis of VAF. For example, VAF is a rare anatomical variation of the vertebral artery. With a history of trauma, the probability of being diagnosed with VAD is 7 times higher than that of VAF in the clinical setting of acute trauma. 5 In addition, VAF also needs to be identified from vertebral artery duplication, which refers to the phenomenon that two vessels of different origin merge into one during their course, 9 and its color Doppler blood flow performance is almost the same as that of VAF. During the dynamic tracking scan, it is also necessary to further identify the starting points of the two vessels.

There is still conflict about the clinical significance of VAF. Some scholars believe that VAF is an accidental discovery with no obvious pathological or clinical consequences. However, there are also literature reports that VAF is prone to be accompanied by other vascular malformations, including intracranial aneurysms, and arteriovenous malformations, 10 which may be related to the medial defects of the vascular wall at both ends of the fenestrated segment and hemodynamic changes, and indicates that the abnormal histological composition of the fenestrated blood vessels may make the blood vessels susceptible to serious pathological consequences. However, VAF may be less fragile and may warrant intervention in patients whose symptoms are suspected to be related to the malformation. A 70-year-old woman with suspected dizziness associated with VAF was reported to have an improvement in her symptoms after surgical correction of the kinked origin of the vertebral artery. 11 There are also reports showing that VAF patients suffer from cerebellar infarction, but whether the cause is related to VAF requires further study. 12 In summary, when imaging examinations reveal vertebral artery anatomic variations (such as VAF), other possible vascular abnormalities should be identified or excluded.

Currently, the “gold standard” for diagnosing VAF is digital subtraction angiography and computed tomography angiography. However, ultrasound examination, as the main screening tool, occupies a core position. Through Doppler examination, blood flow velocity and its related parameters can be monitored intuitively, thereby objectively evaluating changes in vascular morphology and hemodynamics.
